# Participatory development of innovation and implementation strategy – an action-oriented approach

**DOI:** 10.1186/s12913-026-14420-6

**Published:** 2026-03-25

**Authors:** Miae Lee, Lesley A. Anderson, Emilia Piltonen, Abha Maheshwari, Julian L. Griffin, Seshadri S. Vasan

**Affiliations:** 1 NHS Grampian, Research & Development, Foresterhill House Annexe, Aberdeen, AB25 2ZB UK; 2 Interdisciplinary Institute, University of Aberdeen, Crombie Annexe, Aberdeen, AB24 3TS UK; 3 School of Medicine, Medical Sciences and Nutrition, University of Aberdeen, Aberdeen, AB25 2ZD UK; 4 NHS Grampian, Aberdeen Maternity Hospital, Cornhill Road, Aberdeen, AB25 2ZL UK; 5 The Rowett Institute of Nutrition and Health, University of Aberdeen, Aberdeen, AB25 2XD UK; 6 School of Medical and Health Sciences, Edith Cowan University, Joondalup, WA 6027 Australia

**Keywords:** Participatory methods, Stakeholder engagement, Implementation strategy, Healthcare innovation, Interdisciplinary collaboration, Digital health, Workforce development, NHS

## Abstract

**Background:**

Healthcare and academic institutions face growing challenges in strategic planning due to rapid advances in medicine and technology, alongside fiscal and workforce constraints that limit traditional consultation. Participatory approaches offer a way to integrate diverse stakeholder perspectives under these constraints, generating contextually relevant strategies that can indicate whether current directions are appropriate or whether priorities have been overlooked.

**Methods:**

A structured, time-limited participatory workshop was conducted at the 10th Grampian Research Conference (June 2025), that brought together National Health Service (NHS) staff, academia, industry and patients and public communities. Participants engaged in 14 parallel roundtable discussions, with contributions captured on posters and Post-it notes, collecting 148 written annotations. Data were analysed using rapid thematic and content analysis, supplemented by strategic frameworks including Strengths, Weaknesses, Opportunities, and Threats (SWOT/TOWS), and Easy Wins, to identify and prioritise actionable strategies.

**Results:**

Five core themes emerged: (1) access to healthcare and services, (2) patient-centred care and partnership, (3) digital health and service delivery innovation, (4) data access, integration, and governance, and (5) workforce development and culture. SWOT analysis identified strengths in telemedicine, interdisciplinary student training, and patient and public involvement, alongside weaknesses in fragmented data, referral tracking, and workforce pressures. TOWS matrix produced strategy-oriented recommendations such as AI-enabled scheduling, remote monitoring, and transparent referral systems. Easy Wins framework assessment highlighted immediate, low-cost improvements including identifiable NHS caller identification, automated text message reminders, updated informational videos and multilingual materials.

**Conclusion:**

By combining participatory outputs with structured strategy tools, this action-oriented approach demonstrated a resource-efficient model for adaptive planning. The findings align with and extend current national health policy frameworks, offering a replicable approach for institutions aiming to obtain meaningful stakeholder engagement despite fiscal and temporal constraints. Moreover, it provides a blueprint for health systems worldwide to accelerate transformation and deliver patient-centred care in an era of unprecedented change.

**Clinical trial number:**

Not applicable.

**Supplementary Information:**

The online version contains supplementary material available at 10.1186/s12913-026-14420-6.

## Background

Healthcare organisations face mounting pressure to adapt strategies in response to rapidly evolving scientific, technological, and societal changes. Advances in genomics, artificial intelligence, and data science are reshaping models of care, while the pace of clinical practice updates increasingly demands rapid integration into everyday care delivery. Traditional five-year strategic planning cycles commonly used in hospitals, universities, and research institutes, struggle to remain relevant and often fail to keep pace with shifting priorities and emerging innovations [1–3]. At the same time, clinicians, researchers, patients, and other stakeholders operate under significant time constraints and workload pressures, limiting their ability to engage in lengthy or repeated consultation processes [4, 5]. This tension underscores a critical implementation challenge: designing engagement processes that are responsive, efficient, and inclusive while remaining feasible within fiscal and temporal constraints.

Participatory approaches, including co-production and co-design, have emerged as promising methods for developing health interventions and strategies that are contextually relevant and more likely to be adopted in practice [6, 7]. Collaborative research and co-design approaches can enhance implementation feasibility and policy relevance by involving diverse stakeholders, from clinicians and academics to patients and the public, in shaping priorities and solutions [8–10]. However, participatory processes are resource-intensive, often requiring multiple workshops, facilitated by team-building exercises, or in-depth stakeholder assessments [11]. In publicly funded systems such as the National Health Service (NHS) Scotland, where resources and workforce capacity are constrained, there is a need for scalable alternatives that retain the benefits of inclusivity while operating within limited time and budget [12].

Engagement mechanisms also face methodological challenges. Open consultations and feedback systems may disproportionately attract individuals with particularly strong experiences, potentially limiting representativeness [13]. Large-scale surveys can broaden participation but often require substantial investment and may lack contextual depth needed to inform implementation planning [13]. Traditional qualitative research methods (including in-depth interviews, focus groups, and structured questionnaires) can generate rich insights but are often time-intensive, resource-heavy, and difficult to implement rapidly in operational settings. As a result, healthcare organisations often oscillate between two extremes: rigorous qualitative studies that are methodically robust but slow and costly, or engagement events that generate broad themes without structured analysis or clear translation into actionable strategies. This gap highlights the need for pragmatic engagement approaches that balance methodological rigour with operational feasibility. Proportionate strategies that aim for analytic sufficiency rather than exhaustive representation may offer a practical compromise, enabling organisations to capture diverse stakeholder perspectives while generating actionable insights within constrained time and resource environments.

Insights from the Science of Team Science (SciTS) further emphasise the importance of structured interdisciplinary collaboration when addressing complex healthcare challenges [14]. Evidence from translational research contexts suggests that diverse teams perform most effectively when interactions are intentionally structured to promote shared understanding and coordinated output [15]. However, many team science models such as Belbin’s team role theory and Institute for Healthcare Improvement (IHI) working styles assume stable, resourced collaboration rather than brief, open-participation engagements [16, 17]. This highlights the need for pragmatic, structures capable of harnessing interdisciplinary expertise within time-limited, real-world settings.

Implementation science offers useful tools for addressing this challenge. Frameworks such as Strengths, Weaknesses, Opportunities and Threats (SWOT) analysis and its strategic extension, the Threats, Opportunities, Weaknesses and Strengths (TOWS) matrix, can be adapted to participatory contexts to translate stakeholder perspectives into actionable strategies [18]. Similarly, the concept of low cost, high impact “easy wins” resonate with the need to deliver visible improvements while larger scale reforms are underway [3, 12]. Applying these structured but flexible methods to participatory workshops may therefore provide a feasible pathway for generating strategy-oriented insights that are both practical and aligned with health system priorities.

**Fig. 1:**
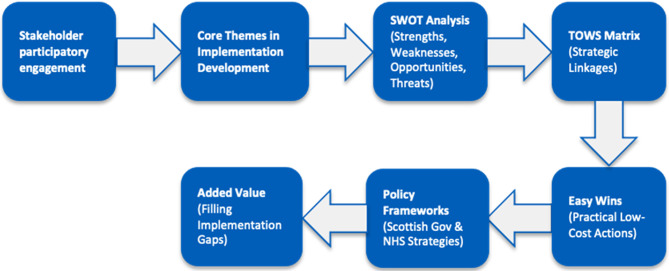
Integrating participatory outputs with strategic planning tools in healthcare implementation

Against this backdrop, we examined whether a structured, time-limited participatory action-oriented workshop format could generate implementable strategies to guide healthcare innovation in Scotland. Grounded in implementation science principles, we tested whether brief, interdisciplinary discussions supported by systematic capture and analysis of outputs could identify both long term priorities and immediate easy wins (Fig. 1). We further assessed how these insights align with, extend, or highlight gaps in existing NHS Scotland policies, including *Realistic Medicine* [19], the *Digital Health and Care Strategy* [20], *Scotland’s AI Strategy* [21], and the *Triple Helix* priorities of NHS-academia-industry collaboration [22].

## Methods

### Rationale

This study was situated within the context of the 10th Grampian Research Conference (27–28 June 2025, Northeast Scotland). The event was designed as a participatory implementation workshop followed by qualitative analysis to identify barriers, facilitators, and system-level priorities for healthcare innovation. The approach uses a validated, rapid qualitative method in health services research to generate actionable insights within constrained timeframes, prioritising analytic sufficiency over exhaustive exploration [23, 24]. The event convened NHS Scotland staff, academics, industry partners, patients, public contributors to explore adoption of evidence-based and emerging innovations.

### Study design and participants

A total of 197 individuals registered for the two-day conference. The morning programme on Day 1 featured six five-minute plenary presentations highlighting healthcare innovations and lived patient experiences, followed by a panel discussion and parallel roundtable session. Presentation topics included drone healthcare technology to improve equity and access, home testing initiatives, the use of artificial intelligence (AI) in cancer screening and pathway redesign, AI-enabled app for reliable health information, and a young patient’s journey navigating NHS services.

The roundtable component comprised 14 concurrent tables, each accommodating approximately 8–10 participants. Because attendees arrived and joined the conference at different times, these figures reflect approximate group sizes rather than the total number of registrants. NHS participants were identified by a show of hands, and while no tables were NHS-only, one academic-only table was asked to integrate with others to support interdisciplinary dialogue. Based on observation by the convenor (LAA) and conference organiser (SSV), most tables were filled, and participants were actively engaged, contributing diverse perspectives across clinical, academic, industry, patient, and public roles.

### Data collection

Parallel roundtable discussions were structured around three guiding questions aligned with implementation research constructs:

1. What’s a key challenge or unmet need in healthcare?
2. What’s an innovative idea that could address it?
3. Who needs to be involved to make it work?

All contributions were generated simultaneously during the structured roundtable session and overseen by conference facilitators and convenors (LAA, JG, AM, and SSV). Participants documented their ideas in real time on Post-it notes or wrote directly onto table posters. At the conclusion of the session, 14 posters, one from each roundtable, containing participant contributions were collected. High-resolution photographs of each poster were taken immediately after collection to create a digital record of the written responses. The posters were publicly displayed in the conference tea and coffee break area until the following morning, allowing cross-table visibility and reflection. No additional written contributions were solicited after the facilitated session.

Following the event, all handwritten responses were entered verbatim into Microsoft^®^ Excel (Version 16.100.4) for analysis. As no audio recordings were made, the dataset consisted solely of written poster annotations. Digital images were cross-checked against the physical posters during data analysis to ensure completeness and accuracy.

### Data analysis and synthesis

We employed a multi-method analytic approach integrating thematic analysis [25] with conventional and summative content analysis [26]. Two research assistants (ML and EP), blinded to participants and not present at the conference, entered and reviewed the dataset to ensure familiarity and minimize bias.

Thematic analysis was first conducted to explore participant perspectives in depth [25]. ML performed an initial review of all posters, followed by an independent review by EP. Post-it notes and directly written annotations were coded line by line, grouped into categories, and iteratively refined into broader themes that captured shared concerns and priorities across tables. Near duplicate entries were retained, as all contributions were generated concurrently during the session, and no exact duplicates were identified. Conventional content analysis was used to ensure these themes remained grounded in the raw data, while summative content analysis quantified the frequency of key words and concepts, highlighting the salience of specific challenges and innovations [26].

In addition, each poster was analysed using a SWOT framework (ML and EP in tandem) to identify strengths, weaknesses, opportunities, and threats related to healthcare innovation. Discrepancies in interpretation were discussed and resolved under lead supervision (LAA, SSV) to ensure consistency. ML then consolidated the poster-level analyses into a single overall SWOT summary and synthesised this into an action-oriented TOWS matrix. This involved iterative reviews and discussions with LAA and SSV to explore strategic relationships between internal capabilities and external conditions, and to support development of actionable strategies [18]. In addition, we identified “easy wins”, low-cost and high-impact improvements aligned with phased implementation principles [12].

### Rigor and credibility

Analytic rigor was ensured through independent coding by two researchers (ML and EP), with oversight from the lead research team (LAA, JG, SSV). Codes and themes were iteratively refined through discussion into a shared codebook using an inductive approach. Discrepancies in coding and interpretation (e.g., handwriting legibility or category assignment) were resolved through consensus. Content analysis continued until all annotations were categorized and no new categories emerged. Given the brief, written nature of the data, the study aimed for analytic sufficiency rather than formal data saturation. Weekly reviews of coding, theme development, and SWOT/TOWS synthesis with clinical and academic team members (LAA, JG, SSV) reinforced analytic credibility and the translational relevance of the findings.

### Ethical considerations

Participation in discussions was voluntary, contributions were anonymized prior to transcription and analysis, no identifying information was retained in the dataset. The North of Scotland Research Ethics Committee confirmed that no ethical concerns were identified, and there were no objections to the publication of the study findings.

### Reception and participation

The roundtable format appeared to be well received as an interactive, inclusive component. The convenor (LAA) circulated among tables to encourage participation and ensure all voices were represented. Contributions were voluntary, and attendees joined discussions at their own pace.

## Results

### Participant overview

The roundtable format engaged a diverse range of stakeholders, including NHS staff, academic researchers, industry representatives, patients, and external partners. Fourteen roundtables were convened, each comprising approximately 8–10 participants, and collectively generating 14 posters containing 148 annotations. Posters were displayed from the afternoon session through the following morning, allowing all participants to view the outputs.

Registration for Day 1 (Table 1) reflects the delegates who checked-in, including late-arrivals. The session took place in the main hall, where each table had 10 chairs. Organisers monitored the hall to ensure that tables were mostly or fully occupied; therefore, participation was estimated at approximately 8–10 individuals per table. Precise participant counts for each roundtable were not recorded, as the organisers aimed to promote an organic discussion rather than a tightly controlled format. A few participants stepped out briefly during the session (e.g., to answer phone calls or have short conversations) but they were observed to return to their tables promptly; therefore, this was not considered a major confounder. Table 1 provides an illustrative overview of stakeholder representation among registrants. All de-identified materials generated during the session formed the dataset for rapid qualitative analysis.

**Table 1:** Illustrative overview of day 1 registrants: characteristics and stakeholder representation

Job Category *	Example Roles	*n*	%
Research (NHS & University)	Research Fellow, PhD Student, Research Nurse, Researcher	49	28.0%
Healthcare	General Practitioner, Clinical Consultant, Psychiatrist, Physiotherapist	40	22.9%
Academic	University Professor, Lecturer, Reader	18	10.3%
R&D department	Research Coordinator, Project Manager, Ethics, Quality Assurance	15	8.6%
Executive/ Management	Network Manager, Chief Officer	15	8.6%
External stakeholder	Industries, Government, Businesses	11	6.3%
Data/Digital/ IT	Data Coordinator, Data analyst, Tech	9	5.1%
Non-R&D (NHS)	NHS Librarian, Healthcare Chaplain, Educator, Champion, Support Manager	8	4.6%
Public/PPI	Volunteer, PPI, Student, Spouse, Chef	6	3.4%
Other University stakeholders	Faculty Development, University Staff	4	2.3%

* Note: Categories reflect participants’ self-reported primary role. “Research (NHS & University)” includes university- or healthcare- based research fellows, nurses, and students engaged in research. “Healthcare Professionals” covers frontline clinical staff. “Academic” refers to university-affiliated teaching or research faculty. “R&D Department” or Research & Development includes NHS staff working in research coordination or administration. “Executive/ Management” represents leadership roles across sectors. “External Stakeholder” includes representatives from industry, government, or business organizations. “Data/Digital/IT” covers data and technology professionals supporting research or healthcare systems. “Non-R&D (NHS)” includes NHS staff in support roles outside research. “Public/PPI” refers to patient and public contributors, including volunteers and community participants. “Other University Stakeholders” includes university staff in non-research roles. Convenors and conference organisers were excluded from these categories. Small categories (< 5%) were retained to represent diversity of participants

## Thematic analysis: core implementation priorities

Thematic analysis identified five overarching themes, with illustrative examples provided in Table 2. Each theme included sub-themes that captured participants’ priorities and recommendations for strengthening NHS service delivery and strategic planning.

## Theme 1: Access to healthcare and services

- **Service Accessibility and Availability**: Participants highlighted the need to enhance timely access to healthcare services through flexible appointments, telemedicine, home testing, visible GP specialties, and efficient care pathways to reduce delays.
- **Equitable Access**: Ensuring fair access was a strong priority, with participants emphasising the need to remove cultural, linguistic and bias-related barriers.
- **Patient Access and Transparency**: Many valued the need to view their health records, referrals, and appointments directly, noting that digital tools could help patients track care in real time and navigate the NHS pathway more efficiently.

## Theme 2: Patient-centred care and partnership

- **Patient-Centred Care**: Participants emphasized placing patients at the centre of innovation by prioritizing their needs, coordinating care across specialities, centralizing health records, and designing digital systems that simplify navigation care pathways.
- **Patient Empowerment with Clear Communication**: Clear, consistent communication was seen as essential to empower decision-making and self-management, while reducing anxiety. Suggestions included recognizable NHS caller ID numbers, digital reminders, transparent updates on care pathways, and easy tools for appointment management (e.g., cancel, reschedule, or request contact with a healthcare professional).
- **Community and Population Health**: A community-based approach was valued for addressing age-related needs, fostering social innovation, and promoting prevention through health education, local support, and coordinated services.

## Theme 3: Digital health & services delivery innovation

- **Automation and Artificial Intelligence (AI)**: Participants noted that potential of AI and automation to improve clinical efficiency across admissions, triage, decision support, documentation, scheduling, reminders and wait time management, while emphasizing the need to monitor and address algorithmic bias.
- **Digital Health and Technology Advancement**: Expanding digital access and advancing technological solutions (i.e., drones, digital apps) were seen as opportunities to improve communication, streamline care, and extend healthcare accessibility to a wider community.
- **Infrastructure and Resources**: Modern, reliable IT and physical infrastructure were identified as critical for enabling digital healthcare advancements. Priorities included reducing firewall barriers, investing in system upgrades, and supporting service design that improves data access and hospital operations.

## Theme 4: Data access, integration, & governance

- **Interoperability and System Coordination**: Participants stressed the importance of standardized, interoperable systems that facilitate communication between services, and enable real-time information sharing, and support open data initiatives for research and service delivery.
- **Data Driven Research and Decision Support**: Strengthening data collection, quality, relevance, and integration across the NHS was seen as key to supporting research, continuous quality improvement, and evidence-informed decision making, with a focus on scalable, cost-effective, high-impact solutions.
- **Governance and Policy Alignment**: Effective innovation was seen as dependent on clear governance frameworks and supportive policies, including automated information governance guidance, open data access, and cross-sector collaboration to ensure responsible technology adoption.

## Theme 5: Workforce development & culture

- **Education and Recruitment**: Participants highlighted the need to prepare the future healthcare workforce by embedding digital literacy, research capacity, and communication skills in early medical education, alongside creating innovative training pathways and strengthening recruitment.
- **Training and Workforce Support**: Upskilling current staff to develop a digitally skilled, AI-enabled workforce was emphasized as a priority. Reducing administrative burden through technology and providing ongoing training and support were viewed as essential for workforce retention.
- **Culture**,** Leadership**,** and Collaboration**: A culture of innovation and technology adoption requires engaged leadership and interdisciplinary collaboration across patients, clinicians, industry, and government. Participants noted the importance of overcoming risk aversion, fostering cross-team communication, and embedding innovation into organizational strategies and funding models.

**Table 2:** Core themes and subthemes with examples of participant annotations

Themes	Sub-themes	Examples		
Access to Healthcare & Services	Service Accessibility and Availability	*“Need access to timely NHS.”*	*“Evening access to health appointments"*	*“Clinic waiting times. Text reminders use of AI. Flexible appointment bookings*,* chose appointments on app?”*
	Equitable Access	*“More equitable access to population-based health - cultural and language sensitive education"*	*“Health inequalities - race and gender bias (within) various path(s)”*	*“Language barriers in symptom description. Also (require) cultural interpretation*,* not just translation"*
	Patient Access and Transparency	*“App for patients - where are you in NHS pathway"*	*“Patient access to results on secure app. Patients need to take more responsibility for own care/ pathway.”*	*“Difficult in patients accessing their own data.”*
Patient-Centred Care and Partnership	Patient-Centred Care	*“Inability to prioritise patient backlog*,* especially for referrals"*	*“Biggest barrier in innovation is not innovating in areas that patients see is a priority. Need proper long term patient involvement and buy in"*	*“Patients - get them onboard innovation.”*
	Patient Empowerment with Clear Communication	*“Reduce patient anxiety by introducing communication app"*	*“Clearly listing GP specialities on website so you) can be seen by someone who is an expert on your issue.”*	*“Self-diagnosis - need recognised NHS resource information to grab and send to GP. Upgrade NHS information videos.”*
	Community and Population Health	*“Health education to the public"*	*“Many single households. Can we encourage more co-living? Communities of joint activities.”*	*“Not enough support groups.”*
Digital Health & Service Delivery Innovation	Automation and Artificial Intelligence	*“Automating admissions and clinics - automated summaries and medical scribes automation.”*	*“AI agent automations assistance given financial constraints for any adequate staffing.”*	*“Possibly use AI system to analyse all patient data from all sources for better recognition/traits.”*
	Digital Health and Technology Advancement	*“NHS Google that is accepted in GP and A + E.”*	*“Extend drone tech.”*	*“Digital bookings by patients via app.”*
	Infrastructure and Resources	*“Dysfunctional IT in hospital.”*	*“Bring IT in NHS Grampian up to speed and reduce firewalls.”*	*“Upgrade NHS information videos.”*
Data Access, Integration & Governance	Interoperability and System Coordination	*“Data linkage.”*	*“Enhanced health record centralisation and linking*,* and accessed by patients via apps*,* dashboards etc.”*	*“Open data initiative and national digital platforms.”*
	Data Driven Research and Decision Support	*“Transition from evidence to real world practise.”*	*“Constant evaluation.”*	*“Co-production service users - clinicians and research (interdisciplinary).”*
	Governance and Policy Alignment	*“Data protection issues and information governance (IG).”*	*“Digital information governance and AI service operators working together to find solutions.”*	*“Patient data privacy and security.”*
Workforce Development & Culture	Education and Recruitment	*“More employment.”*	*“Recruiting future workforce.”*	*“Clinical student introduction course to know better about technology and research.”*
	Training and Workforce Support	*“Upskilling operations managers (OMs) - training education.”*	*“Digital and AI enabled workforce.”*	*“Reduce workload*,* e.g. mental health and radiology.”*
	Culture, Leadership, and Interdisciplinary collaboration	*“Innovative managers = innovative teams and projective time.”*	*“Collaboration with industry and clinicians*,* and patient and public involvement.”*	*“Risk averse culture.”*

### Summative content analysis: frequency and salience of ideas

**Fig. 2:**
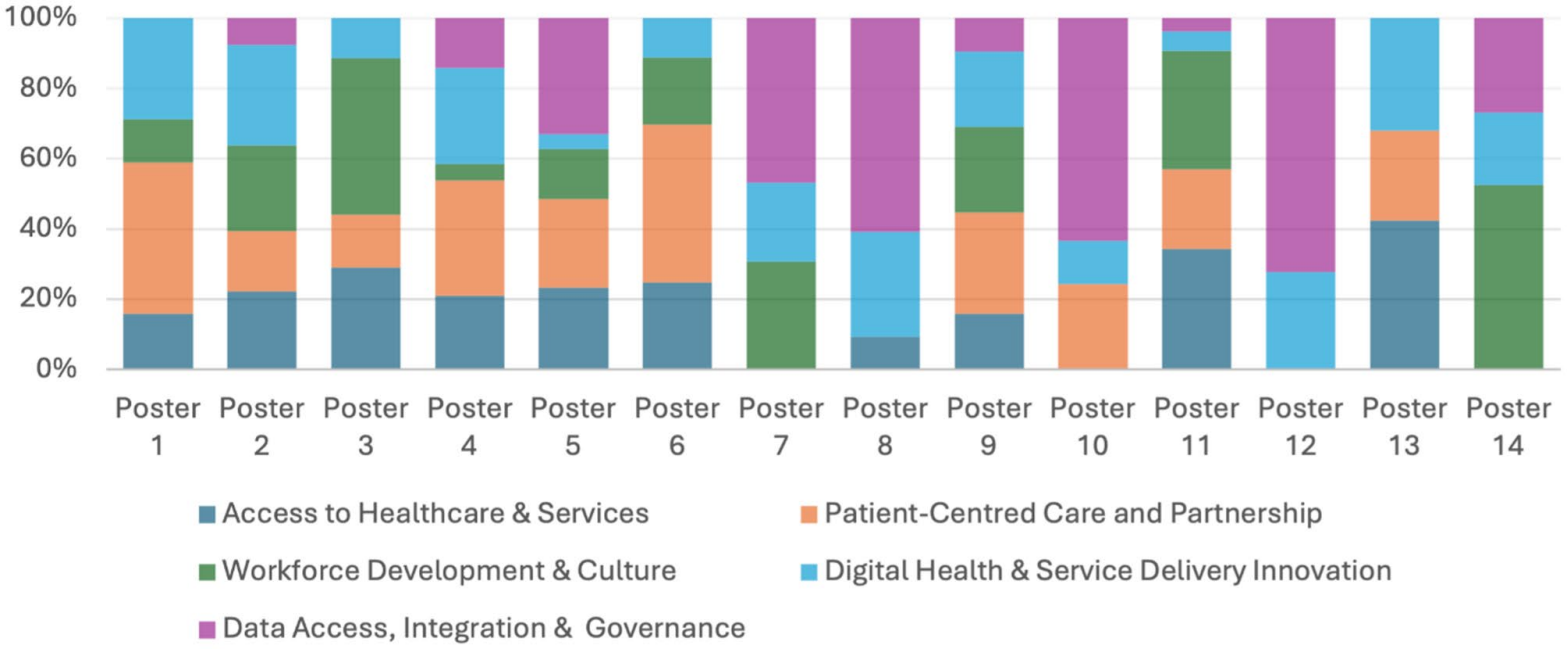
Theme distribution across posters

Summative content analysis identified the most frequently occurring concepts across the dataset, providing insight into both the breadth of issues raised and their relative prominence (Fig. 2). Annotation reflecting multiple themes were counted once within each applicable theme. Therefore, individual annotations may contribute to more than one thematic category. Percentages represent the proportion of total coded annotations (*n* = 211) assigned to each theme.

The most frequently represented theme was Digital Health and Service Delivery Innovation (26.5%), appearing in every roundtable, followed by Access to Healthcare and Services (24.2%), Data Access, Integration, and Governance (20.4%), Workforce Development & Culture (15.6%), and Patient Centred Care and Partnership (13.3%). Frequently occurring terms included *data*,* access*,* patients*,* AI*, and *innovation*, reflecting shared priorities across stakeholder groups (*Supplemental Material*). Less frequent but highly salient terms such as *workforce*,* culture*,* infrastructure*,* and governance* clustered within specific tables, highlighting areas of concentrated stakeholder concern.

Together, these findings underscore the dual importance of widely shared priorities, such as digital transformation and access, alongside more targeted issues (e.g., workforce culture, infrastructure gaps) raised by specific groups.

### SWOT analysis: strategic landscape of innovation

Each poster was analysed using a SWOT framework to identify internal strengths and weaknesses and external opportunities and threats, summarized in Table 3.

- **Internal strengths** included the emerging implementation of new technology in NHS Grampian and the northeast islands, such as telemedicine, e-consults, and integrated care networks, as well as ongoing research in drone-assisted medication delivery. Additional strengths included interdisciplinary education for clinical students and Patient and Public Involvement (PPI), referring to the meaningful involvement of patients and the public as partners in shaping research, policy, and service delivery. Together, these demonstrate a growing readiness to embed digital tools and patient-centred approaches to service delivery.
- **Internal weaknesses** highlighted persistent system-level barriers, including access and capacity challenges, fragmented data systems, and communication gaps between services and patients. These underscore the need for greater interoperability and service integration to translate innovation into measurable improvements.
- **External opportunities** reflected broader system trends, including digital and AI-enabled transformation, workforce development, and enhanced patient engagement through data-driven care. Seizing these opportunities will require alignment with national policy priorities and sustainable investment strategies.
- **External threats** encompassed resource pressures, technology and data risks, environmental barriers such as air traffic control regulations for drone delivery, and uncertainty in policy or funding landscapes. These external constraints highlight the importance of risk mitigation and equitable implementation planning.

Overall, this analysis provided a structured overview of the strategic landscape for NHS innovation, highlighting both enablers and constraints that must be addressed for effective implementation.

**Table 3:** Stakeholder-derived SWOT analysis summary

Internal Strength	Internal Weaknesses
**Expanded Access to Care**: Telemedicine, e-consults, and NHS care networks, and on-going research in drone delivery extend advanced healthcare to remote patients and smaller hospitals.	**Access and Capacity Challenges**: Delays in care, limited appointment availability, GP/service gaps, workforce shortages, and late-hour access issues
**Education and Interdisciplinary Training**: Clinical students gain interdisciplinary training, gaining strong technology and research skills	**Data and Information Gaps**: Fragmented systems, poor interoperability, inconsistent data quality, limited patient access to information, and slow translation of research into practice.
**Patient and Public Involvement**: PPI initiatives enable patients and the public to actively shape research and healthcare services, ensuring their perspectives are central to decision-making.	**Communication and Coordination Issues**: Breakdowns between services, teams, and patients; poor referral tracking; and inadequate integration between primary and secondary care
**External Opportunities**	**External Threats**
**Digital & AI-Enabled Transformation**: Leveraging AI, digital platforms, telemedicine, and interoperable systems to streamline care and enhance patient access.	**Resource & Capacity Pressures**: Aging population pressures, staff shortages, burnout, and overloaded services.
**Workforce Development & Efficiency**: Upskilling, flexible staffing, innovative job planning, and interdisciplinary collaboration to optimize care delivery.	**Technology & Data Risks**: Slow adoption, infrastructure lags, cyber/privacy risks, digital exclusion, and AI algorithm bias.
**Patient Engagement & Data-Driven Care**: Empowering patients through access to records, personalized communication, co-designed services, and data-informed pathways	**Policy**,** Funding & Cultural Barriers**: Limited funding, governance blocks, policy restrictions, and resistance to change

### TOWS matrix: translating findings into strategy

The TOWS synthesis mapped internal strengths and weaknesses against external opportunities and threats to generate strategy-oriented recommendations, summarized in Table 4.

- **(SO) ‘Maxi-Maxi’ Strategy**: Internal strengths (i.e., existing resources that expand access to care, interdisciplinary training, and PPI) can be harnessed to drive digital and AI-enabled transformation, scaling telemedicine, unifying care through shared records, and empowering patients with personalized, user-friendly portals.
- **(ST) ‘Maxi-Mini’ Strategy**: These strengths also help mitigate resource and capacity pressures by deploying tech-skilled students to accelerate technology adoption and strengthening patient engagement.
- **(WO) ‘Mini-Maxi’ Strategy**: Internal weaknesses (including access and capacity challenges, fragmented data, and communication breakdowns) were addressed through strategies such as exploring AI-driven scheduling, remote monitoring, flexible staffing, upskilling, and transparent referral tracking.
- **(WT) ‘Mini-Mini’ Strategy**: External threats (including resource constraints, technology and data risks, and policy or funding barriers) were countered with recommendations to develop demand forecasting and invest in governance, interoperability, data quality, AI validation, and integrated care pathways.

Overall, the TOWS analysis translated broad insights into strategy-oriented directions for NHS digital, workforce, and patient-centred innovation.

**Table 4:** TOWS matrix linking internal and external factors to proposed strategies

	External Opportunities	External Threats
**TOWS matrix**	*Digital & AI-Enabled Transformation*	*Resource & Capacity Constraints*
	*Workforce Development & Efficiency*	Technology & Data Risks
	*Patient Engagement & Data-Driven Care*	*Policy, Funding & Cultural Barriers*
**Internal Strength**		
*Expanded Access to Care*	(1) Expand healthcare access with telemedicine, AI-driven triage, and interdisciplinary training for future clinicians.	(1) Deploy telehealth to reduce staff shortage and aging population pressures.
*Education and Interdisciplinary Training*	(2) Unify care through shared records and interoperability.	(2) Leverage tech-skilled students to offset slow adoption risks.
*Patient and Public Involvement*	(3) Empower patients with personalized, co-designed, data-driven portals.	(3) Strengthen patient engagement systems against policy resistance and digital exclusions.
**Internal Weaknesses**		
*Access and Capacity Challenges*	(1) Cut wait time with AI scheduling, remote monitoring, and virtual care.	(1) Prevent bottlenecks with demand forecasting and workforce optimization.
*Data and Information Gaps*	(2) Boost capacity with flexible staffing and upskilling.	(2) Mitigate risks through governance interoperability, data quality, and AI validation.
*Communication and Coordination Issues*	(3) Strengthen referrals and coordination through portal tracking and data-driven tools.	(3) Advance integrated care pathways with system investment and policy alignment.

### Easy wins: immediate, low-cost implementation actions

Stakeholders identified practical, low-cost “easy wins” to accelerate implementation (Table 5). Recommendations included standardising automated text message reminders, enabling digital appointment cancellations, and providing language-inclusive materials and updated NHS informational videos to promote equity. Piloting online support groups in high-demand areas such as dementia, chronic care, and mental health, alongside broader public health education campaigns, was suggested to reduce isolation and service pressures. Incremental improvements to patient engagement tools (i.e., adding GP specialty listings, secure medical record access, and referral tracking) were highlighted as ways to empower patients and improve communication. Deploying students in tech-enabled roles, including triage support, digital training, and patient onboarding, was recommended to help alleviate workforce pressures while building future-ready skills.

Together, thematic analysis, SWOT/TOWS strategy mapping, and easy wins collectively provide a comprehensive, stakeholder-driven actionable framework for strengthening healthcare service delivery through digital innovation, patient-centred care, workforce development, and equitable access.

**Table 5:** “Easy Wins”: Illustrative implementation considerations for patient-identified priorities

Easy Wins	Description	Implementation Considerations
Cutting no-shows	Implement automated text reminders, digital cancellation options, and recognisable NHS caller ID to reduce missed appointments and wasted clinic slots. Standardise systems already used in some clinics.	**Stakeholders**: Digital transformation and service operations functions **Equity**: Provide non-digital alternatives (phone, mail) for digitally excluded patients **Resources**: Low (uses existing infrastructure) **Timeline**: Short term **Example indicators**: Reduction in DNA (Did Not Attend) rates, increase in advance cancellations, patient-reported trust in appointment communications
Inclusive communication	Expand language inclusivity measures (translations, plain language materials) and update NHS informational videos for clarity, providing a low-cost, high-impact step toward equity.	**Stakeholders**: Communications and equality & diversity teams **Equity**: Prioritise high-need languages, include British Sign Language and easy read formats, involve patients in reviewing materials **Resources**: Low-Medium **Timeline**: Short term **Example indicators**: Number of materials translated, uptake of translated resources, patient comprehension scores (survey)
Virtual support and education	Pilot online support groups for dementia, chronic illness, or mental health to reduce isolation and ease service demand.	**Stakeholders**: Primary care networks and mental health services **Equity**: Offer hybrid options, monitor participation by age, geography, and socioeconomic status. **Resources**: Medium **Timeline**: Short - medium term (pilot) **Example indicators**: Enrolment and attendance rates, reduction in non-urgent GP visits, patient-reported wellbeing or isolation scores.
Smarter patient portals	Standardise and expand patient portals across clinics and simplify rollout for GP practices, with the goal of wider implementation. Over time, portals could expand to include secure access to medical records and referral tracking to help patients monitor their care pathway with incremental improvements co-designed with patients to ensure usability.	**Stakeholders**: Digital health and IT support teams **Equity**: Ensure accessibility for older adults and people with disabilities, provide assisted digital support. **Resources**: Medium **Timeline**: Short - medium term (implementation roadmap) **Example indicators**: Percentage of practices onboarded, portal activation and usage rates, reduction in administrative queries.
Future-ready workforce	Deploy clinical students in supervised, tech-enabled roles (e.g., triage support, digital onboarding) to support service delivery while building future workforce skills through structured placements or “earn-as-you-learn” pilots.	**Stakeholders**: Workforce development teams and academic partners **Equity**: Ensure equitable access to placements, avoid replacing paid staff, support rural placements **Resources**: Medium **Timeline**: Medium term (pilot) **Example indicators**: Number of students deployed, staff time saved (hours/month), student satisfaction and retention into NHS roles.

Note: Examples are illustrative and provided to demonstrate how patient-identified priorities might translate into potential practical actions. Descriptions may reflect activities already underway, areas under consideration, or illustrative approaches not currently planned within the next five years by the organisation. They do not represent organisational commitments or formal implementation plans

## Discussion

This study demonstrates that structured, time-limited participatory workshops can efficiently capture strategic priorities and implementable solutions, even within the constraints of limited resources and workforce pressures. By engaging NHS staff, academic researchers, industry representatives, patients, and public contributors, the roundtable process produced a coherent set of implementation strategies that complement and extend national health policies.

Thematic analysis revealed five interconnected themes: (1) access to healthcare and services, (2) patient centred care and partnership, (3) digital health and service delivery innovation, (4) data access, integration, and governance, and (5) workforce development and culture. These findings align strongly with existing frameworks and reinforce existing Scottish policies, but also identify critical operational details, particularly around patient-facing digital tools, communication equity measures, and student workforce innovation, that are not yet fully articulated in national strategies.

The *Triple Helix* model emphasises cross-sector collaboration: the multi-stakeholder approach adopted here reflects this principle and underscores the value of interdisciplinary co-design in building an innovation ecosystem that can deliver practical, evidence-informed solutions [22]. The principles of *Realistic Medicine* and the *Population Health Framework* were also evident: participants prioritised transparent care pathways, shared decision-making, and community-based initiatives, and suggested that embedding these values within digital platforms could enhance engagement and patient satisfaction [19, 27]. Scotland’s *AI Strategy* was mirrored in the recognition of AI and automation as enablers of efficiency and decision support [21], coupled with calls for safeguards to mitigate algorithmic bias and ensure equitable access [20]. These perspectives align with NHS digital governance priorities and the NHS Grampian R&D Strategy, which advocates embedding research and innovation into routine practice [12, 28].

The SWOT analysis identified system strengths (e.g., telemedicine, interdisciplinary training, and PPI) alongside persistent system-level weaknesses (e.g., fragmented data, capacity constraints, communication breakdowns). While these broadly align with the transformative aims of Scotland’s *Digital Health and Care* and *AI Strategies* [20, 21], participants also pinpointed barriers at a more granular level, notably inconsistent referral tracking and the slow translation of research into practice. These issues are not explicitly addressed in national strategies but are beginning to be tackled through initiatives such as the first phase of the new public-facing *Digital Front Door* programme, a personalized digital healthcare service, and *Data Safe Haven* platforms [2, 29]. For example Aberdeen’s local *Grampian Data Safe Haven* (DaSH), one of five data safe havens in Scotland, securely links and provides access to pseudo-identified health data for ethically governed research [29].

Participants also identified PPI, supported through the *Public Involvement Network*, which provides regular updates and opportunities for public involvement in research and service design, as a key strength [30]. However, the broader concept of Patient and Public Involvement, Engagement, and Participation (PPIEP), as defined by the National Institute for Health and Care Research (NIHR), distinguishes between three related but distinct activities: involvement (active partnership in shaping research and services), engagement (communication and outreach to inform and raise awareness), and participation (taking part in research studies) [31, 32]. While PPI is embedded and routinely required, Patient and Public Engagement (PPE) activities remain limited by budget constraints, with only one event held in the past two years, a challenge shared across wider NHS health boards [28]. Notably, participation remains strong, exemplified by initiatives such as Widening Access to Trials in Care Homes (WATCH) project, through which NHS Grampian is leading national efforts to involve care home residents in vaccine research, strengthen informed consent practices, and contribute to UK-wide guidance in this area [33].

The TOWS matrix, combined with “Easy Wins”, translated workshop insights into actionable strategies. Strengths such as telemedicine and student training pipelines can be leveraged to offset workforce shortages and resource pressures, aligning with the *Triple Helix* principle of joint NHS, academia, and industry investment. Meanwhile, weaknesses in access and data systems could be framed as opportunities for AI-driven scheduling and remote monitoring. These are both being addressed in the *Digital Front Door* programme, supporting the Scottish Government’s *Population Health framework* by promoting equity, efficiency, and accountability [2, 27].

Stakeholders also identified several low-cost, high impact “Easy Wins” to improve healthcare experiences. Table 5 presents illustrative implementation considerations demonstrating how these priorities could potentially be operationalised within healthcare systems. While a complementary Strengths, Opportunities, Aspirations, Results (SOAR) analysis provided a strengths-based, aspirational perspective with measurable outcomes for implementation, the TOWS framework and “Easy Wins” were prioritised for their stronger emphasis on actionable strategies within real-world system constraints [27, 34]. The SOAR analysis is presented in the Supplemental Material.

Taken together, the findings show that participatory workshops can serve as a practical method of generating both long-term strategies and immediate operational actions. They bridge the gap between high-level policy and frontline realities, producing outputs that are actionable, resource-conscious, and aligned with implementation science principles of contextual relevance, feasibility, and stakeholder ownership. By highlighting specific operational gaps, patient-facing referral transparency, low-cost communication equity measures, and structured student involvement, this study contributes directly to the implementation literature and offers NHS Scotland actionable levers for accelerating innovation adoption.

### Study limitations

This study has several limitations that should be considered when interpreting the findings. Conference presentations delivered immediately prior to the roundtables may have introduced a priming effect, potentially shaping the direction of discussion. In future events, this could be mitigated by reordering sessions so that roundtables occur before presentations. Participation and seating were voluntary and not systematically recorded, limiting insight into table composition and possible clustering of similar perspectives. This was inevitable in a conference setting, where attendees typically choose where to sit and are not expected to participate in tightly controlled focus groups. Although patients and public contributors were present, their representation was proportionally lower than NHS and academic stakeholders, which may have influenced the balance of perspectives captured in the discussions. We plan to address this in future conferences by actively reaching out to PPI groups. Data were derived from brief handwritten poster annotations rather than audio-recorded discussion, which limited depth and precluded assessment of participation dynamics within groups. Finally, findings derive from a single regional conference within NHS Scotland, which limits generalisability, and replication in routine service settings is warranted. These constraints reflect the practical realities of conducting structured stakeholder engagement within a conference setting and should be considered when interpreting the scope and transferability of the findings.

### Benefits and reflection for future

Despite these limitations, the conference successfully fostered strong audience engagement and created opportunities for meaningful dialogue and knowledge-sharing across sectors. Structured opportunities for networking and discussion were incorporated throughout, enabling participants to reflect on system needs and exchange perspectives. Discussions explored policies needed within the NHS, incorporating diverse perspectives from stakeholders across NHS staff, academia, industries, and patients. This collaborative approach aligns closely with the principles of *Realistic Medicine*, particularly its emphasis on shared decision-making, reducing unwarranted variation, and ensuring that care is person-centred and value driven [19]. Overall, the conference was well-received and offered valuable insights to inform future initiatives.

Reflections on the conference format highlighted several lessons for future events. The use of scribes, rather than reliance on participant handwriting, may improve the accuracy and completeness of recorded feedback. Encouraging participants to sit with people they don’t know or to spread out across departments, where feasible, can promote diverse perspectives without recording seating assignments, and participants generally follow such guidance. In addition, scheduling speaker presentations after round-table sessions may help prevent undue influence on participants, reducing the risk of shaping ideas in advance. Collectively, these adjustments may enhance engagement, inclusivity, and diversity in future conferences.

## Conclusion

Participatory workshops provide a feasible, resource-efficient mechanism for generating implementation strategies in time-limited, resource-constrained health systems. Combining thematic, content, SWOT/TOWS analyses yielded actionable insights, identifying key challenges, enablers, and operational gaps to accelerate adoption, foster equity, and improve patient experience. These findings show the value of participatory, action-oriented methods for aligning policy, practice, and patient priorities, and for producing both immediate and longer-term strategies to support sustainable innovation adoption.

## Supplementary Information

Below is the link to the electronic supplementary material.

Supplementary Material 1

## Data Availability

The datasets analysed during the current study are available from the corresponding author on reasonable request.

## Abbreviations

AI: Artificial Intelligence
NHS: National Health Service
SWOT: Strengths, Weaknesses, Opportunities, Threats
TOWS: Threats, Opportunities, Weaknesses, Strengths
ID: Identification
R&D: Research and Development
IT: Information Technology
PPI: Patient and Public Involvement
DaSH: Data Safe Haven
PPE: Patient and Public Engagement
UK: United Kingdom
SOAR: Strengths, Opportunities, Aspirations, Results
