# Supplementary material for: Participatory development of innovation and implementation strategy – an action-oriented approach
Source: BMC Health Serv Res. 2026 Mar 25;26:626. doi: 10.1186/s12913-026-14420-6 (PMC13137527; doi:10.1186/s12913-026-14420-6)
Supplement: Supplementary file 1 — Supplementary Material 1 [file 12913_2026_14420_MOESM1_ESM.docx]

|  | Supplemental Material _ **Standards for Reporting Qualitative Research (SRQR)*** |  |
| --- | --- | --- |
|  | <http://www.equator-network.org/reporting-guidelines/srqr/> |  |
| **Title and abstract** | | **Page(s).** |
|  | **Title** - Concise description of the nature and topic of the study Identifying the study as qualitative or indicating the approach (e.g., ethnography, grounded theory) or data collection methods (e.g., interview, focus group) is recommended | Pg 1 |
|  | **Abstract** - Summary of key elements of the study using the abstract format of the intended publication; typically includes background, purpose, methods, results, and conclusions | Pg 2-3 |
| **Introduction** | |  |
|  | **Problem formulation** - Description and significance of the problem/phenomenon studied; review of relevant theory and empirical work; problem statement | Pg 4-6 |
|  | **Purpose or research questio**n - Purpose of the study and specific objectives or questions | Pg 6 |
| **Methods** | |  |
|  | **Qualitative approach and research paradigm** - Qualitative approach (e.g., ethnography, grounded theory, case study, phenomenology, narrative research) and guiding theory if appropriate; identifying the research paradigm (e.g., postpositivist, constructivist/ interpretivist) is also recommended; rationale** | Pg 7-9 |
|  | **Researcher characteristics and reflexivity** - Researchers’ characteristics that may influence the research, including personal attributes, qualifications/experience, relationship with participants, assumptions, and/or presuppositions; potential or actual interaction between researchers’ characteristics and the research questions, approach, methods, results, and/or transferability | Pg 8-10 |
|  | **Context** - Setting/site and salient contextual factors; rationale** | Pg 7-8 |
|  | **Sampling strategy** - How and why research participants, documents, or events were selected; criteria for deciding when no further sampling was necessary (e.g., sampling saturation); rationale** | Pg 7 and Pg 11 |
|  | **Ethical issues pertaining to human subjects** - Documentation of approval by an appropriate ethics review board and participant consent, or explanation for lack thereof; other confidentiality and data security issues | Pg 10 and Pg 29 |
|  | **Data collection methods** - Types of data collected; details of data collection procedures including (as appropriate) start and stop dates of data collection and analysis, iterative process, triangulation of sources/methods, and modification of procedures in response to evolving study findings; rationale** | Pg 8-9 |
|  | **Data collection instruments and technologies** - Description of instruments (e.g., interview guides, questionnaires) and devices (e.g., audio recorders) used for data collection; if/how the instrument(s) changed over the course of the study | Pg 7-8 |
|  | **Units of study** - Number and relevant characteristics of participants, documents, or events included in the study; level of participation (could be reported in results) | Pg 7-10 and *Table 1* |
|  | **Data processing** - Methods for processing data prior to and during analysis, including transcription, data entry, data management and security, verification of data integrity, data coding, and anonymization/de-identification of excerpts | Pg 8-9 |
|  | **Data analysis** - Process by which inferences, themes, etc., were identified and developed, including the researchers involved in data analysis; usually references a specific paradigm or approach; rationale** | Pg 9-10 |
|  | **Techniques to enhance trustworthiness** - Techniques to enhance trustworthiness and credibility of data analysis (e.g., member checking, audit trail, triangulation); rationale** | Pg 10 |
| **Results/findings** | |  |
|  | **Synthesis and interpretation** - Main findings (e.g., interpretations, inferences, and themes); might include development of a theory or model, or integration with prior research or theory | Pg 11-23 |
|  | **Links to empirical data** - Evidence (e.g., quotes, field notes, text excerpts, photographs) to substantiate analytic findings | *Tables 2, 3, 4, 5* |
| **Discussion** | |  |
|  | **Integration with prior work, implications, transferability, and contribution(s) to the field -** Short summary of main findings; explanation of how findings and conclusions connect to, support, elaborate on, or challenge conclusions of earlier scholarship; discussion of scope of application/generalizability; identification of unique contribution(s) to scholarship in a discipline or field | Pg 24-27 |
|  | **Limitations** - Trustworthiness and limitations of findings | Pg 27 |
| **Other** | |  |
|  | **Conflicts of interest** - Potential sources of influence or perceived influence on study conduct and conclusions; how these were managed | Pg 30 |
|  | **Funding** - Sources of funding and other support; role of funders in data collection, interpretation, and reporting | Pg 30 |
|  | *The authors created the SRQR by searching the literature to identify guidelines, reporting standards, and critical appraisal criteria for qualitative research; reviewing the reference lists of retrieved sources; and contacting experts to gain feedback. The SRQR aims to improve the transparency of all aspects of qualitative research by providing clear standards for reporting qualitative research. |  |
|  | **The rationale should briefly discuss the justification for choosing that theory, approach, method, or technique rather than other options available, the assumptions and limitations implicit in those choices, and how those choices influence study conclusions and transferability. As appropriate, the rationale for several items might be discussed together. |  |
|  | **Reference:** |  |
|  | O'Brien BC, Harris IB, Beckman TJ, Reed DA, Cook DA. **Standards for reporting qualitative research: a synthesis of recommendations.** *Academic Medicine*, Vol. 89, No. 9 / Sept 2014  DOI: 10.1097/ACM.0000000000000388 |  |
|  |  |  |

Supplemental Material _ *Word cloud highlighting the 50 most frequently used words from the poster quotations**
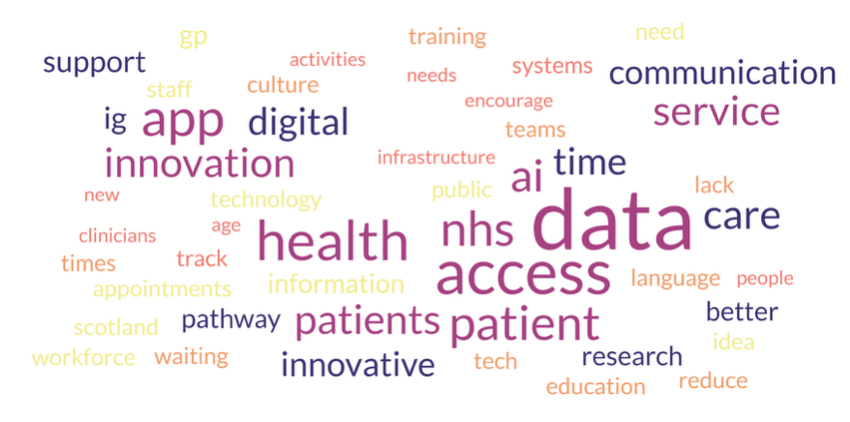
*

Supplemental Material _ SOAR analysis

| **Strength (What we are doing well)** | **Opportunities (Where we can grow)** |
| --- | --- |
| **Expanded Access to Care:** Telemedicine, e-consults, and NHS care networks, and on-going research in drone delivery extend advanced healthcare to remote patients and smaller hospitals. | **Digital & AI-Enabled Transformation:** Leveraging AI, digital platforms, telemedicine, and interoperable systems to streamline care and enhance patient access. |
| **Education and Interdisciplinary Training:** Clinical students gain interdisciplinary training, gaining strong technology and research skills | **Workforce Development & Efficiency:** Upskilling, flexible staffing, innovative job planning, and interdisciplinary collaboration to optimize care delivery. |
| **Patient and Public Involvement:** PPI initiatives enable patients and the public to actively shape research and healthcare services, ensuring their perspectives are central to decision-making. | **Patient Engagement & Data-Driven Care:** Empowering patients through access to records, personalized communication, co-designed services, and data-informed pathways |
| **Aspirations (What we want to become)** | **Results (Measurable outcomes)** |
| **Timely and Equitable Access:** Ensure patients receive seamless, prompt care, with minimal delays and equitable access across all settings, positioning us as a national leader in rural and remote innovation (telehealth, drone logistics, virtual care). | **Sustainable, Resilient Care Delivery:** Consistent achievement of national access and waiting time standards, year-on-year reduction in referral-to-treatment delays, sustained reduction in staff vacancy and turnover rates, improvement in workforce wellbeing and burnout. |
| **Seamless Collaboration Across Care:** End fragmentation and achieve seamless communication between primary, secondary, and community services. | **Secure, Inclusive, and Effective Digital Systems**: Widespread adoption of interoperable shared care records across services, increase in patient access and use of digital health tools, reduction in disparities in digital access across populations. |
| **Real-Time Patient Data Navigation:** Develop fully interoperable high-quality, timely data, enabling patients to track referrals, appointments, and care progression easily. | **Enabling Policy & Adaptive Funding Environment:** Dedicated and sustained investment in innovation and digital transformation, regular launch and evaluation of new care models and pilots, shortened time from policy approval to implementation, routine reporting of quality and equity performance metrics. |
